# OPPRESSIVE EXPERIENCES AMONG DEAF DIVERSE SENIOR CITIZENS

**DOI:** 10.1093/geroni/igz038.930

**Published:** 2019-11-08

**Authors:** Audrey K Frank

**Affiliations:** Gallaudet University, Washington, District of Columbia, United States

## Abstract

None of the limited literature on deaf seniors focuses on their experiences of oppression. There is an article that demonstrates that not many mental health professionals have the skills to work with deaf seniors. Obstacles have been noted in the literature among deaf people in general terms of being oppressed with employment, doctors, education, family discrimination, stereotyping, stigmas and cultural conflicts because they are deaf. The obstacles facing deaf seniors had not been specifically explored before this research. The shift attention to deaf seniors is needed in order to make their experiences known and at the same time their lives, values, and strengths need to be understood and recognized. Ninety-one deaf seniors from five states aged between 50 years and 93 years were interviewed to describe their recent experience on oppression. They shared their common experiences on oppression such as lack of communication with their doctors, hearing co-workers, and family members, struggling to get promoted at work, and being left out in the neighborhood. According to the deaf seniors, the community did not acknowledge or accommodate the special needs they had as deaf seniors. Their detailed descriptions provide consistent evidence that supports the importance of cultural awareness for medical and mental health professionals. The professionals are to enhance better understanding of experiences among deaf seniors. This lack of awareness highlights the need for research about deaf seniors’ experiences of oppression and for research on what professionals should know about the special needs of this population.

